# Mucinous tumor arising in a giant sacrococcygeal teratoma: A rare case report

**DOI:** 10.1097/MD.0000000000008759

**Published:** 2017-11-27

**Authors:** Fengtian Zhang, Xiaolong Yu, Jin Zeng, Min Dai

**Affiliations:** Department of Orthopedics, The First Affiliated Hospital of Nanchang University, Artificial Joints Engineering and Technology Research Center of Jiangxi Province, Nanchang, Jiangxi, China.

**Keywords:** adult, mucinous, sacrococcygeal, teratoma, tumor

## Abstract

**Rationale:**

Teratomas, which most frequently affect adult females, are the most common type of germ cell tumor, it always comprises derivatives of at least 2 germ layers. The most common site of primary teratomas is the ovary. Sacrococcygeal teratomas (SCTs), which are exceedingly rare in adults, are generally found in newborns or children.

**Patient concerns:**

A 39-year-old woman presented to our clinic with a 1-year history of gradually aggravated difficulty in micturition and defecation, and a tumor in her right buttock present since birth. Appropriate preoperative examinations showed a large (15.6 cm × 12.2 cm × 30.0 cm) multicystic SCT.

**Diagnoses:**

Histologic examination confirmed a mucinous tumor arising in a giant SCT.

**Interventions:**

Abdominoperineal rectal resection was performed.

**Outcomes:**

The patient recovered well and was discharged on day 33 of admission.

**Lessons:**

We report the first case of a mucinous tumor arising in an SCT, in which the teratoma presented mature tissue elements derived only from the endodermal germ layer (keratinous debris).

## 1 Introduction

Teratomas, which most frequently affect adult females, are the most common type of germ cell tumor. The most common site of primary teratomas is the ovary, followed by the testis, anterior mediastinum, retroperitoneum, and sacrococcygeal area.^[1]^ Sacrococcygeal teratomas (SCTs), which are exceedingly rare in adults, are generally found in newborns or children, having an incidence of 1 in 30,000 to 43,000 live births, with a male-to-female ratio of 3 to 4:1.^[2]^

Teratomas always comprise derivatives of at least 2 germ layers, including the mesoderm, endoderm, and ectoderm, and are deemed as tumors. Teratomas have the potential to imitate many neoplasms of various organs and to copy almost all tissues of the human body, in a very flexible way. Especially, pituitary teratomas are very rare, with only very few cases reported.^[3–5]^

Mucinous tumors are present in 2% to 11% of mature ovarian cystic teratomas, and 3% to 8% of mucinous ovarian tumors are associated with teratomas.^[6]^ Currently, it is generally accepted that primary low-grade appendiceal mucinous neoplasms can metastasize to the ovaries and subsequently give rise to mucinous ascites/pseudomyxoma peritonei, which may also result from the intraabdominal spread of an appendiceal mucinous neoplasm.^[7–10]^ However, to our knowledge, there is currently no report about a mucinous tumor arising in an SCT.

We here present an extremely rare case of a mucinous tumor arising in a large, mature, multicystic SCT in a 39-year-old woman. The tumor consisted of 3 main cysts: 2 cysts containing numerous thin-walled, mucin-filled cysts, and a single cyst containing only keratinous debris, which was composed of derivatives of the endodermal germ layer.

## 2 Case presentation

A 39-year-old woman presented with a tumor in her right buttock, which was present since birth. The tumor was gradually increasing in size but had not been previously treated. She visited our clinic due to a 1-year history of gradually aggravated difficulty in micturition and defecation, characterized by an inability to void without external pressure applied. Meanwhile, she also experienced pain that made it impossible for her to remain still in any position. The tumor was bulky and solid on palpation and could be felt from the sacrum and coccyx. On digital rectal examination, the finger could not get above the extraluminal cystic mass in the posterior wall of the rectum. However, no obvious abnormality in the rectal mucosa was noted.

Routine laboratory tests revealed alpha-fetoprotein (AFP) 1.48 ng/mL (normal range, 0–7.0), carcinoembryonic antigen (CEA) 42.68 ng/mL (normal range, 0–6.5 ng/mL), carbohydrate antigen 19-9 (CA19-9) 58.40 U/mL (normal range, 0–27 U/mL), and carbohydrate antigen 72-4 (CA72-4) 45.12 U/mL (normal range, 0–6.9 U/mL).

**Figure 1:**
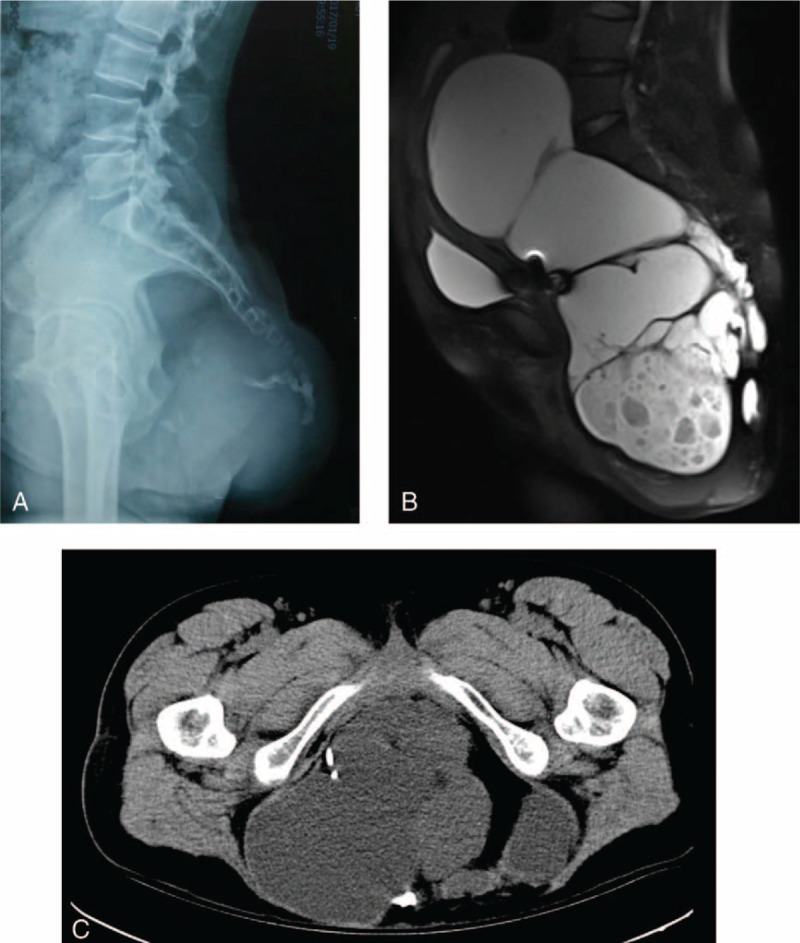
Imaging findings. A large presacral tumor can be seen on plain radiographs, which had invaded the coccyx (A). Enhanced magnetic resonance imaging showed a presacral heterogeneous tumor containing 3 multiloculate cystic areas, one of which presented an enhanced cystic lesion with a solid small nodule component (B). Abdominopelvic computed tomography showed a presacral heterogeneous tumor, 15.6 cm × 12.2 cm × 30.0 cm in size, containing numerous thin-walled and solid components with calcification and fat (C).

A large presacral tumor could be seen on plain radiographs, which had invaded the coccyx (Fig. 1A). Abdominopelvic computed tomography demonstrated a presacral heterogeneous tumor, 15.6 cm × 12.2 cm × 30.0 cm in size, containing numerous thin-walled and solid components with calcification and fat (Fig. 1B and C). Enhanced magnetic resonance imaging showed a presacral heterogeneous tumor containing 3 multiloculate cystic areas, one of which comprised an enhanced cystic lesion with a small nodule solid component. Based on these findings, the patient was diagnosed with SCT.

**Figure 2:**
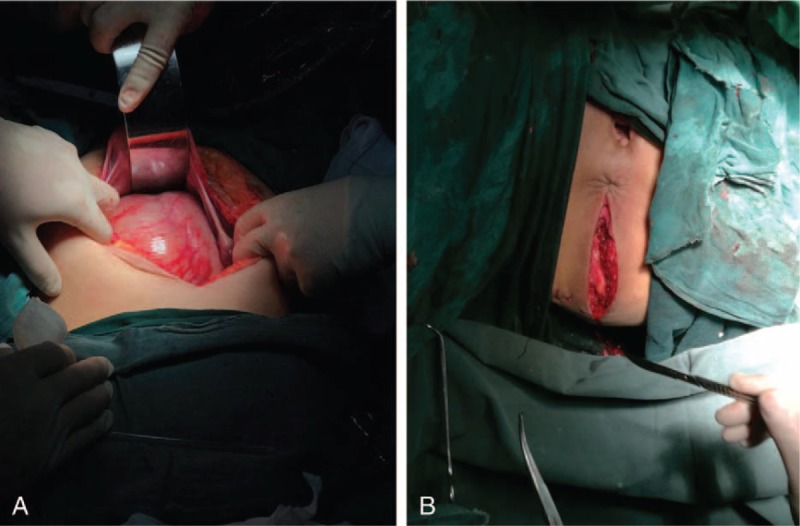
Operative approach. The abdominoperineal approach was performed to remove the tumor (A and B).

**Figure 3:**
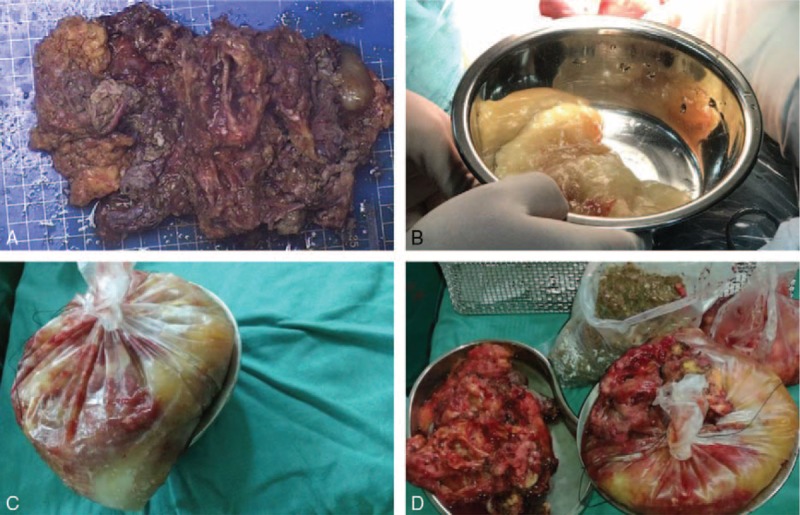
Gross findings. The cystic walls (A). Mucinous jelly-like material was noted in one cyst (B and C). The mucinous jelly-like material, cystic walls, and a solid gray-white element can be seen (D).

Abdominoperineal rectal resection was performed (Fig. 2A and B). On laparotomy, there was no evidence of peritoneal dissemination, although the tumor was found to have invaded the rectum. Accordingly, we advised the family members of the patient that permanent colostomy should be performed; however, her family members refused this procedure. Hence, we kept the rectum under the premise of removing as much of the tumor as possible. On sectioning, the tumor showed 2 cysts that contained only numerous thin-walled, mucin-filled cysts, and a single cyst that contained a solid gray-white element only (Fig. 3A–D).

**Figure 4:**
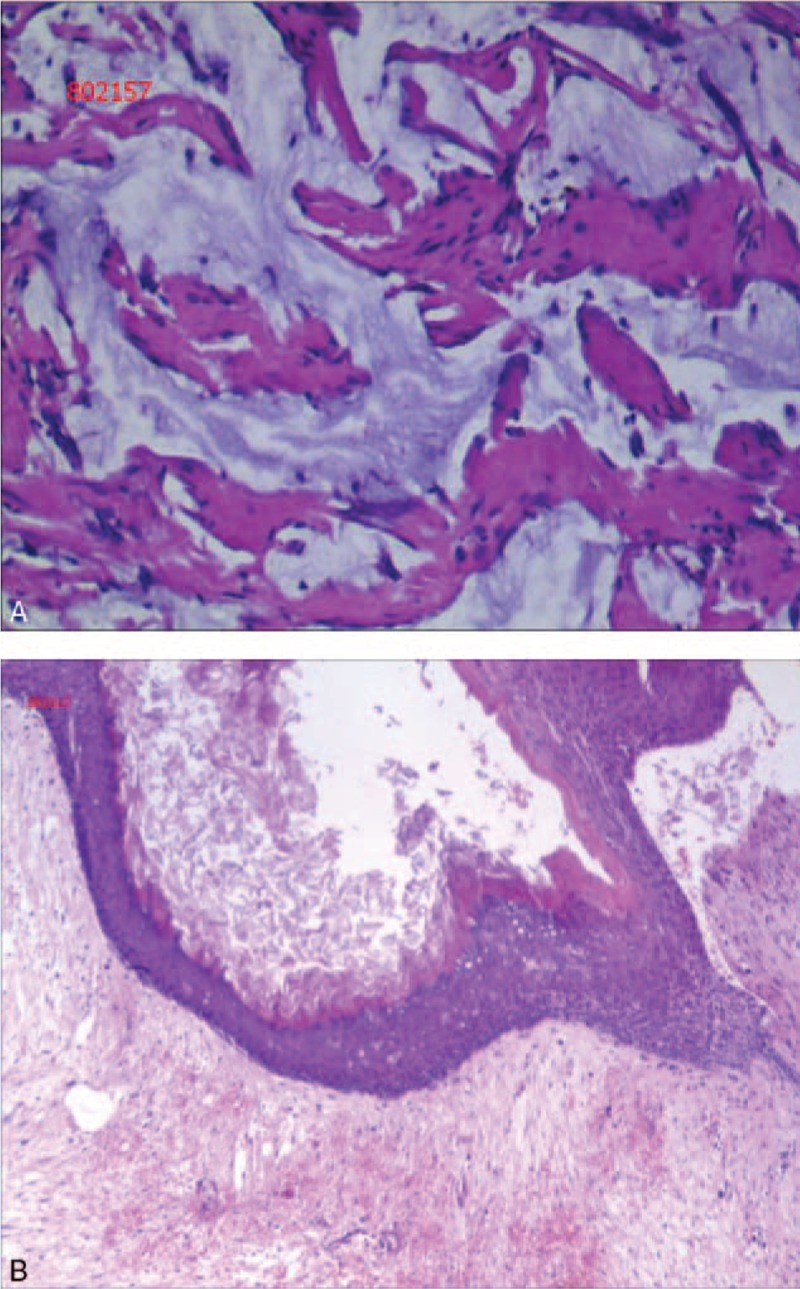
Histology findings. Invading mucin can be seen around the fibrous tissue, whereas no cells were found (hematoxylin and eosin stain, ×400) (A). The histology findings revealed a mature differentiated teratoma containing an endodermal germ layer with keratinous debris (hematoxylin and eosin stain, ×100) (B).

Pathologic assessment (Fig. 4A and B) of the fresh gross surgical specimen revealed that the solid gray-white element consisted of keratinous debris (Fig. 4B). On the other hand, the mucinous jelly-like material showed invading mucin only, and no cells were observed (Fig. 4A).

The patient recovered well and was discharged on day 33 of admission. She did not receive any adjuvant therapy. The patient is currently under clinical and imaging follow-up to identify any future recurrence.

## 3 Discussion

The most common site of mucinous tumors is the appendix, followed by the ovaries^[10,11]^; however, rare cases of these tumors occurring in other various organs, including the testes^[12]^ and retroperitoneum^[13,14]^ have been reported. Mucinous tumors are reportedly present in 2% to 11% of mature ovarian cystic teratomas, and 3% to 8% of mucinous ovarian tumors are associated with teratomas.^[6]^ On the other hand, this is, to our knowledge, the first report of a mucinous tumor arising in an SCT. It is generally accepted that the origin of mucinous tumors is usually the ovaries or appendix^[10,11]^; however, in the present case, the appendix and ovaries were all normal. There have also been some reports of these tumors originating in various other organs such as the gallbladder and bile ducts, stomach, pancreas, colon, fallopian tube, uterine corpus, urachus, urinary bladder, breast, and lung.^[6]^ However, the mucinous jelly-like material observed in our patient showed invading mucin only, and no cells were seen (Fig. 4A). Moreover, the patient had had a tumor in her right buttock since birth. Accordingly, we consider the present tumor as a primary SCT with invading mucinous discharge from the cells of the cyst. In other words, the cells in the cysts were likely actively proliferating, suggesting a poor prognosis if left untreated.

Teratomas always comprise derivatives of at least 2 germ layers, including the mesoderm, endoderm, and ectoderm, and have the potential to imitate many neoplasms of various organs and to copy almost all tissues of the human body. Especially, pituitary teratomas are very rare, with only very few cases reported.^[3–5]^ In the present case, the teratoma presented mature tissue elements derived only from the endodermal germ layer (keratinous debris) (Fig. 4B), which is very rare.

SCTs are classified according to the presence of intraabdominal or presacral extension. Type I SCT tumors are predominantly external and have only a minimal presacral component; Type II tumors are predominantly external but have a definite intraabdominal extension; Type III tumors have a small external component and are mostly located in the abdomen, or comprise predominantly a pelvic mass that extends into the abdomen; while Type IV lesions are entirely presacral with no external component.^[2,15,16]^ In the present case, the mass could be seen at the patient's hip, but the predominant mass was pelvic and extended into the abdomen; hence it was classified as SCT type III (Fig. 1A–C). To a certain extent, this classification affects the planning of the surgery; in the present case, we chose to perform abdominoperineal rectal resection.

SCTs can express a diversity of serum tumor markers such as elevated AFP, CEA, CA72-4, and CA19-9^[17–20]^ and these tumor markers are helpful in identifying SCT in clinical practice. Especially, the CA19-9-positive rates in immature, mature, and malignant SCTs have been reported as 57%, 57%, and 48.6%, respectively.^[21]^ In our case, the serum AFP, CEA, CA19-9, and CA72-4 levels were 1.54 ng/mL (normal range, 0–7.0 ng/mL), 42.68 ng/mL (normal range, 0–6.5 ng/mL), 58.40 U/mL (normal range, 0–27 U/mL), and 45.12 U/mL (normal range, 0–6.9 U/mL), respectively. Although the AFP level was within the normal range, we speculate that the reason for this finding was that the tumor had not yet undergone malignant transformation. Postoperatively, the serum AFP, CEA, CA19-9, and CA72-4 levels were 1.48 ng/mL, 4.17 ng/mL, 11.25 U/mL, and 6.35 U/mL, respectively, indicating marked decreases in the CEA, CA19-9, and CA72-4 levels. These results confirm the value of these serologic markers in diagnosing SCT. In addition, a slight decrease in the AFP level was also observed.

Taking into account the spread of the tumor and the risk for recurrence, we deemed it inappropriate to obtain biopsy samples from the rectum and skin of our patient. Instead, we diagnosed SCT and designed the operation plan according to the radiographic investigations. The postoperative pathology confirmed this diagnosis.

The preferred initial treatment modality for SCTs or mucinous tumors includes complete en bloc resection of the tumor, with or without chemotherapy, both in children and adults.^[22,23]^ The surgical approach selected should be based on numerous factors, including the tumor size, location, and invasion to the surrounding organs, and appropriate selection might be the key to successful operation. The main surgical approach generally includes the transabdominal, transsacral, or transperineum approach, or a combination thereof.^[24–27]^ In our case, the abdominoperineal approach was adopted, owing to a suspicion that the tumor had invaded the rectum and due to its large size. On laparotomy, there was no evidence of peritoneal dissemination, although the tumor was found to have invaded the rectum. As the patient's family members refused colostomy, we kept the rectum while removing as much of the tumor as possible. Presently, endoscopic resection of teratomas is also performed in some hospitals; however, this approach is not appropriate for large tumors like in our case.^[28]^

Our case highlights the following points in terms of resection of giant SCTs by the abdominoperineal approach: the specific location of the tumor and the relationship with the surrounding organs should be defined before the operation to select the appropriate surgical approach. When a resection is made at the perineum, it is important to try to avoid the anus, as it is easy to cause infection due to fecal contamination of the incision. Of course, bowel preparation and replacing the wound dressing in a timely manner after urination and defecation postoperatively are also important to avoid infection. In cases of large tumors, in which the residual cavity is large, a drainage tube should be placed in the cavity to reduce the risk of hematoma formation. Lastly, care should be taken not to remove the skin incision suture prematurely; intermittent removal of the suture after more than 2 weeks is recommended to prevent the wounds from reopening.

## References

[1] Sasi W Ricchetti GA Parvanta L Giant mature primary retroperitoneal teratoma in a young adult: report of a rare case and literature review. Case Rep Surg 2014;2014: 930538. 10.1155/2014/930538 PMC4258356 25506459

[2] Ghosh J Eglinton T Frizelle FA Presacral tumours in adults. Surgeon 2007;5:31–8. 17313126 10.1016/s1479-666x(07)80109-0

[3] Saeger W Ebrahimi A Beschorner R Teratoma of the Sellar Region: a case report. Endocr Pathol 2017;DOI 10.1007/s12022-016-9465-0. [Epub ahead of print]. 10.1007/s12022-016-9465-0 28102527

[4] Nishioka H Ito H Haraoka J Immature teratoma originating from the pituitary gland: case report. Neurosurgery 1999;44:644–7. discussion 647–648. 10069602 10.1097/00006123-199903000-00115

[5] Chiloiro S Giampietro A Bianchi A Clinical management of teratoma, a rare hypothalamic-pituitary neoplasia. Endocrine 2016;53:636–42. 26701679 10.1007/s12020-015-0814-4

[6] Motos Mico J Velasco Albendea FJ Ferrer Márquez M [Mucinous tumour in a mature ovarian teratoma: an unusual presentation of pseudomyxoma peritonei]. Cir Esp 2015;93:e69–71. 24120222 10.1016/j.ciresp.2013.04.013

[7] Posabella A Galetti K Engelberger S A huge mucinous cystadenoma of ovarian: a rare case report and review of the literature. Rare Tumors 2014;6:5225. 25002945 10.4081/rt.2014.5225 PMC4083665

[8] Roy S Mukhopadhayay S Gupta M Mature cystic teratoma with co-existent mucinous cystadenocarcinoma in the same ovary—a diagnostic dilemma. J Clin Diagn Res 2016;10:Ed11–3. 10.7860/JCDR/2016/22150.9118 PMC5296442 28208869

[9] Ronnett BM Seidman JD Mucinous tumors arising in ovarian mature cystic teratomas: relationship to the clinical syndrome of pseudomyxoma peritonei. Am J Surg Pathol 2003;27:650–7. 12717249 10.1097/00000478-200305000-00008

[10] Choi YJ Lee SH Kim MS Whole-exome sequencing identified the genetic origin of a mucinous neoplasm in a mature cystic teratoma. Pathology 2016;48:372–6. 27114374 10.1016/j.pathol.2016.02.017

[11] Nemejcova K Dundr P Rosmusová J Sebaceous adenoma arising in mature cystic teratoma of the ovary. Case report. Cesk Patol 2017;53:35–7. 28248120

[12] Kim G Kwon D Na HY Mucinous cystadenoma of the testis: a case report with immunohistochemical findings. J Pathol Transl Med 2017;51:180–4. 28189139 10.4132/jptm.2016.08.30 PMC5357754

[13] Dayan D Abu-Abeid S Klausner JM Primary retroperitoneal mucinous cystic neoplasm: authors’ experience and review of the literature. Am J Clin Oncol 2016;39:433–40. 27258676 10.1097/COC.0000000000000298

[14] Gohda Y Noguchi R Horie T Pseudomyxoma peritonei of a mature ovarian teratoma caused by mismatch repair deficiency in a patient with Lynch syndrome: a case report. BMC Med Genet 2016;17:94. 27938333 10.1186/s12881-016-0356-5 PMC5148915

[15] Altman RP Randolph JG Lilly JR Sacrococcygeal teratoma: American Academy of Pediatrics Surgical Section Survey-1973. J Pediatr Surg 1974;9:389–98. 4843993 10.1016/s0022-3468(74)80297-6

[16] Schropp KP Lobe TE Rao B Sacrococcygeal teratoma: the experience of four decades. J Pediatr Surg 1992;27:1075–8. discussion 1078–1079. 1403540 10.1016/0022-3468(92)90563-m

[17] Ishida C Iwase A Osuka S Serum pentraxin 3 as a possible marker for mature cystic teratomas. Gynecol Endocrinol 2016;32:733–6. 26965297 10.3109/09513590.2016.1157862

[18] Chen C Li JD Huang H [Diagnostic value of multiple tumor marker detection for mature and immature teratoma of the ovary]. Ai Zheng 2008;27:92–5. 18184473

[19] Tjalma WA The value of AFP in congenital cervical teratoma. J Pediatr Surg 2003;38:1846. 14666486 10.1016/j.jpedsurg.2003.08.022

[20] Nanki Y Chiyoda T Kataoka F Elevated preoperative neutrophil: lymphocyte ratio as a preoperative indicator of mature cystic teratoma with malignant transformation. J Obstet Gynaecol Res 2017;43:744–8. 28370831 10.1111/jog.13271 PMC6191647

[21] Dede M Gungor S Yenen MC CA19-9 may have clinical significance in mature cystic teratomas of the ovary. Int J Gynecol Cancer 2006;16:189–93. 16445632 10.1111/j.1525-1438.2006.00284.x

[22] Tuladhar R Patole SK Whitehall JS Sacrococcygeal teratoma in the perinatal period. Postgrad Med J 2000;76:754–9. 11085765 10.1136/pmj.76.902.754 PMC1741825

[23] Khalil BA Aziz A Kapur P Long-term outcomes of surgery for malignant sacrococcygeal teratoma: 20-year experience of a regional UK centre. Pediatr Surg Int 2009;25:247–50. 19184053 10.1007/s00383-009-2329-7

[24] Canelles E Roig JV Cantos M [Presacral tumors. Analysis of 20 surgically treated patients]. Cir Esp 2009;85:371–7. 19423088 10.1016/j.ciresp.2009.01.007

[25] Wishnia SC Rosen JE Hamid MA Management of a presacral teratoma in an adult. J Clin Oncol 2008;26:2586–9. 18487577 10.1200/JCO.2007.15.6034

[26] Nalbanski B Markov D Brankov O [Sacrococcygeal teratoma—a case report and literature review]. Akush Ginekol (Sofiia) 2007;46:41–5. 17469451

[27] Graves CE Idowu O Zovickian J Use of intraoperative lateral pelvic X-ray to localize and ensure coccyx removal during sacrococcygeal teratoma resection. Pediatr Surg Int 2017;33:389–92. 27858188 10.1007/s00383-016-4025-8

[28] Mabuchi Y Ota N Kobayashi A Identical twins with mature cystic teratomas treated with laparoscopic surgery: two case reports. Mol Clin Oncol 2017;6:276–8. 28357110 10.3892/mco.2016.1118 PMC5351738

